# Cortical Thickness Abnormalities in Late Adolescence with Online Gaming Addiction

**DOI:** 10.1371/journal.pone.0053055

**Published:** 2013-01-09

**Authors:** Kai Yuan, Ping Cheng, Tao Dong, Yanzhi Bi, Lihong Xing, Dahua Yu, Limei Zhao, Minghao Dong, Karen M. von Deneen, Yijun Liu, Wei Qin, Jie Tian

**Affiliations:** 1 Life Sciences Research Center, School of Life Sciences and Technology, Xidian University, Xi’an, Shaanxi, China; 2 Information Processing Laboratory, School of Information Engineering, Inner Mongolia University of Science and Technology, Baotou, Inner Mongolia, China; 3 Departments of Psychiatry and Neuroscience, McKnight Brain Institute, University of Florida, Gainesville, Florida, United States of America; 4 Institute of Automation, Chinese Academy of Sciences, Beijing, China; Centre Hospitalier Universitaire Vaudois Lausanne - CHUV, UNIL, Switzerland

## Abstract

Online gaming addiction, as the most popular subtype of Internet addiction, had gained more and more attention from the whole world. However, the structural differences in cortical thickness of the brain between adolescents with online gaming addiction and healthy controls are not well unknown; neither was its association with the impaired cognitive control ability. High-resolution magnetic resonance imaging scans from late adolescence with online gaming addiction (n = 18) and age-, education- and gender-matched controls (n = 18) were acquired. The cortical thickness measurement method was employed to investigate alterations of cortical thickness in individuals with online gaming addiction. The color-word Stroop task was employed to investigate the functional implications of the cortical thickness abnormalities. Imaging data revealed increased cortical thickness in the left precentral cortex, precuneus, middle frontal cortex, inferior temporal and middle temporal cortices in late adolescence with online gaming addiction; meanwhile, the cortical thicknesses of the left lateral orbitofrontal cortex (OFC), insula, lingual gyrus, the right postcentral gyrus, entorhinal cortex and inferior parietal cortex were decreased. Correlation analysis demonstrated that the cortical thicknesses of the left precentral cortex, precuneus and lingual gyrus correlated with duration of online gaming addiction and the cortical thickness of the OFC correlated with the impaired task performance during the color-word Stroop task in adolescents with online gaming addiction. The findings in the current study suggested that the cortical thickness abnormalities of these regions may be implicated in the underlying pathophysiology of online gaming addiction.

## Introduction

As an important period between childhood and adulthood, adolescence is encompassed by alterations in physical, psychological, and social development [1]. The relatively immature cognitive control ability makes this period a time of vulnerability and adjustment and may lead to a higher incidence of affective disorders and addiction among adolescents [2], [3], [4]. As one of the common mental health problems amongst Chinese adolescents, Internet addiction disorder (IAD) is currently becoming more and more serious [5], [6]. Online gaming addiction, as the most important subtype of IAD, had gained more and more attention from the whole world and especially from east Asia, e.g. China and Korea. The adolescents with online gaming addiction spend excessive amount of time playing online games and are unable to control their excessive gaming habits despite detrimental social and emotional consequences, such as declined work performance and academic failure [7], [8], [9], and in extreme cases, even criminal activities [10]. Because of its growing prevalence, IAD and online gaming addiction has drawn scientific attention from academia around the world [5], [6], [7], [8], [9], [11], [12], [13], [14], [15], [16], [17], [18]. Unfortunately, there is currently no standardized treatment for IAD due to the lack of a clear understanding of the mechanisms underlying this disease [12].

To investigate the neural basis of online gaming addiction, emerging neuroimaging studies had been performed and had highlighted functional abnormalities in individuals with online gaming addiction [19]. Based on the abnormal glucose metabolism in the right orbitofrontal cortex (OFC) and the other regions [20] and levels of dopamine D2 receptor availability in the striatum [21] in the online gaming addiction group, the researchers suggested that online gaming addiction may share similar psychological and neurobiological abnormalities with addictive disorders with and without substance. Consistent with this view, Ko et al. identified the neural substrates of craving for online gaming by revealing the activation of several brain regions in response to the gaming cues in the online gaming addiction group, such as the OFC, the anterior cingulated cortex (ACC), the dorsolateral prefrontal cortex (DLPFC) and the parahippocampus [22], [23]. The functional imaging studies had detected the possible neural mechanisms of the online gaming addiction, however, the structural effects of online gaming addiction on the cortical thickness of late adolescence’ brain are not well known [5], [24]. Although the voxel-based morphometry (VBM) method had revealed the gray matter deficits in the ACC, the DLPFC, the OFC, the insula and left lingual gyrus, the supplementary motor area (SMA) and the cerebellum in online gaming addiction individuals [5], [24], this method is especially susceptible to the differences in registration, degree of smoothing, and choice of normalization template [25], [26]. Moreover, to the best of our knowledge, few studies have examined, so far, the cortical thickness abnormalities and its association with the cognitive control impairment in the adolescents with online gaming addiction.

Therefore, cortical thickness measurement method, a more appropriate method than VBM, was employed in the present study to investigate the integrity of cytoarchitecture in the cortex in online gaming addiction group [27], [28]. To interpret the relevance of any cortical thickness abnormalities, the possible behavioral implications of these findings were examined by the correlation analysis between the cortical thickness findings and behavioral measures. Previous studies had revealed the significant correlation between the structural abnormalities and the duration of online gaming addiction [5]. Furthermore, researchers had detected the impaired cognitive control ability in the adolescents with IAD using a color-word Stroop task [29]. Therefore, the behavioral assessments in the present study were duration of the online gaming addiction and the color-word Stroop task performaces. The connection of neuroimaging findings to well-defined behavioral indices that are known to be affected in online gaming addiction would be a further index of the importance of these findings to addiction.

## Methods and Materials

### 2.1 Ethics Statement

All research procedures were approved by the West China Hospital Subcommittee on Human Studies and were conducted in accordance with the Declaration of Helsinki. All participants in our study gave written informed consent.

### 2.2 Participants

According to the modified Young Diagnostic Questionnaire for Internet addiction (YDQ) criteria by Beard and Wolf [17], [30], 165 freshman and sophomore students were screened in eight months. Twenty students with online gaming addiction were filtered out and 18 adolescents with online gaming addiction (12 males, mean age = 19.4±3.1 years, education 13.4±2.5 years) engaged in our study by excluding two left-handed players. Only the individuals with no personal or family history of psychiatric disorders participated in our further study. To investigate whether or not there were any linear changes in the brain structure, the duration of the disease was estimated via a retrospective diagnosis. We asked the subjects to recall their life-style when they were initially addicted to their mainly online game, i.e. World of Warcraft (WOW), which is a massively multiplayer online role-playing game (MMORPG) by Blizzard Entertainment. When playing online game, players need to build avatars in their virtual world and a very large number of players interact with one another within a virtual game world. With 9.1 million subscribers (12 Million at peak) as of August 2012, WOW is currently the world’s most-subscribed MMORPG, and holds the Guinness World Record for the most popular MMORPG by subscribers (http://www.ign.com/articles/2012/10/04/mists-of-pandaria-pushes-warcraft-subs-over-10-million). To guarantee that they were suffering from Internet addiction, we retested them with the YDQ criteria modified by Beard and Wolf. We also confirmed the reliability of the self-reports from the online gaming addiction individuals by talking with their parents via telephone and the roommates and classmates.

Eighteen age- and gender-matched healthy controls (12 males, mean age = 19.5±2.8 years, education 13.3±2.0 years) with no personal or family history of psychiatric disorders also participated in our study. According to previous studies [5], [22], we chose healthy controls who spent less than 2 hours per day on the Internet. The healthy controls were also tested with the YDQ criteria modified by Beard and Wolf to ensure they were not suffering from online gaming addiction. All recruited participants screened were native right-handed Chinese and were assessed by a personal self-report and Edinburgh Handedness Questionnaire. Exclusion criteria for both groups were 1) existence of a neurological disorder evaluated by the Structured Clinical Interview for Diagnostic and Statistical Manual of Mental Disorders, Fourth Edition (DSM-IV); 2) alcohol, nicotine or drug abuse by urine drug screening; 3) pregnancy or menstrual period in women; and 4) any physical illness such as a brain tumor, hepatitis, or epilepsy as assessed according to clinical evaluations and medical records. The Hamilton anxiety scale (HAMA) and the Beck depression inventory-II (BDI) were used to evaluate the emotional states of all participants during the preceding two weeks. More detailed demographic information is given in Table 1.

**Table 1:** Subject demographics for adolescents with online gaming addiction (age range: 17–22 years) and control groups (age ranges: 17–21 years).

Items	Gaming addictionN = 18	ControlN = 18	*P* value
Age (years)	19.4±3.1 (17–22)	19.5±2.8 (17–21)	0.71
Education (years)	13.4±2.5 (12–13)	13.3±2.0 (12–13)	0.64
Duration of internet addiction (months)	34.8±8.5 (24–45)	N/A	N/A
Hours of internet use (/day)	10.2±2.6 (8–15)	0.8±0.4 (1–2)	0.002
Days of internet use (/week)	6.3±0.5 (6–7)	1.6±0.8 (1–3)	0.003
Hamilton anxiety scale	12.4±10.4 (5–21)	6.5±2.9 (1–16)	0.25
Beck depression inventory	11.4±6.8 (3–18)	4.3±2.5 (1–11)	0.004

The education level was matched between the addiction group (education range: 12–13 years) and control group (education range: 12–13 years). The more detailed information, which was described as Mean ± SD (range: min-max), can be found in the table.

### 2.3 Behavioral Data Collection

The color-word Stroop task design was implemented by using E-prime 2.0 software (http://www.pstnet.com/eprime.cfm) according to a previous study [31]. This task employed a block design with three conditions, i.e. congruent, incongruent and rest. Three words, Red, Blue, and Green were displayed in three colors (red, blue and green) as the congruent and incongruent stimuli. During rest, a cross was displayed at the center of the screen, and subjects were required to fix their eyes on this cross without responding. All events were programmed into two runs with different sequences of congruent and incongruent blocks. Each participant was instructed to respond to the displayed color as fast as possible by pressing a button on a Serial Response Box™ with his right hand. Button presses by the index, middle, and ring finger corresponded to red, blue, and green respectively. Participants were tested individually in a quiet room when they were in a calm state of mind. After the initial practice, the behavior data was collected two or three days before MRI scanning.

### 2.4 MRI Data Acquisitions

Magnetic resonance measurements were carried out on a 3-T scanner (Allegra; Siemens Medical System) at the Huaxi MR Research Center, West China Hospital of Sichuan University, Chengdu, China. The high-resolution 3D T1-weighted images were obtained for cortical thickness measurements with the following parameters: TR = 1900 ms; TE = 2.26 ms; flip angle = 90°; in-plane matrix resolution = 256×256; slices = 176; field of view = 256 mm×256 mm; voxel size = 1×1×1 mm. Images were screened by a neurologist for pathological findings.

### 2.5 Imaging Data Analysis

Before the cortical thickness analysis, we had visually checked the quality of raw data for subsequent pipeline. The images with distortion and artifact were excluded. Fortunately, no subject was removed according to the criteria. FreeSurfer 5.0 (http://surfer.nmr.mgh.harvard.edu/) was employed to calculate the cortical thickness from the structural magnetic resonance images. Local cortical thickness was measured on the basis of the difference between the position of equivalent vertices in the pial and gray-white matter surfaces. In brief, cerebral white matter was segmented from the T1-weighted images and the gray-white matter interface was estimated. Topographical defects in the gray-white estimate were fixed, which were then used as the starting point for the deformable surface algorithm search for the pial surface. The surface of the gray-white matter border was inflated, and differences between subjects in the depth of the gyri and sulci were normalized. The reconstructed brain of each subject was deformed and registered to an average spherical surface. To obtain cortical thickness difference maps, the data were smoothed on the surface with a Gaussian smoothing kernel with a full-width half maximum of 10 mm. Due to the fact that the BDI score was significantly different between the two groups, the comparison of regional cortical thickness variations between groups was tested by vertex-by-vertex analysis of covariance (ANCOVA) including BDI as the covariate. To correct for multiple comparisons, *p* maps were thresholded to yield an expected false discovery rate (FDR) of 0.05. Cluster comprising of the vertexes showing significantly different cortical thicknesses between online gaming addiction group and control groups were defined. The average thicknesses of the cluster were extracted and used to calculate the % difference to indicate the magnitude of the effect. To investigate the relationship between cortical thickness findings and online gaming addiction, whole brain correlation analysis between cortical thickness and behavioral assessments (i.e. duration and Stroop task response errors respectively) was introduced in the current study. The peak values of the cluster showing significant correlation with the behavioral information (FDR, *p*<0.05) were extracted and used to calculate the correlation coefficients. In the current study, we focused on the brain regions with significantly different cortical thicknesses between online gaming addiction and control groups.

## Results

Our results demonstrated that the rate of online gaming addiction was about 12.1% in our small sample investigation. According to their self-report of Internet use, the online gaming addiction individuals spent 10.2±2.6 hours per day and 6.3±0.5 days per week on online gaming. Adolescents with online gaming addiction spent more hours per day and more days per week on Internet than the controls (*p*<0.005) (Table 1).

### 3.1 Behavioral Data Results

Both groups showed a significant Stroop effect, where the reaction time was longer during the incongruent than the congruent condition (online gaming addiction: 677.26±75.37 vs 581.19±71.59 and control: 638.32±65.87 vs 548.97±50.59; *p*<0.005). The online gaming addiction group committed more errors than the control group during the incongruent condition (8.56±4.77 vs 4.56±2.93; *p*<0.05), although the response delay measured by reaction time (RT) during the incongruent condition minus congruent conditions was not significantly different between these two groups (98.2±40.37 vs 91.92±45.87; *p*>0.05).

### 3.2 Imaging Data Results

**Figure 1:**
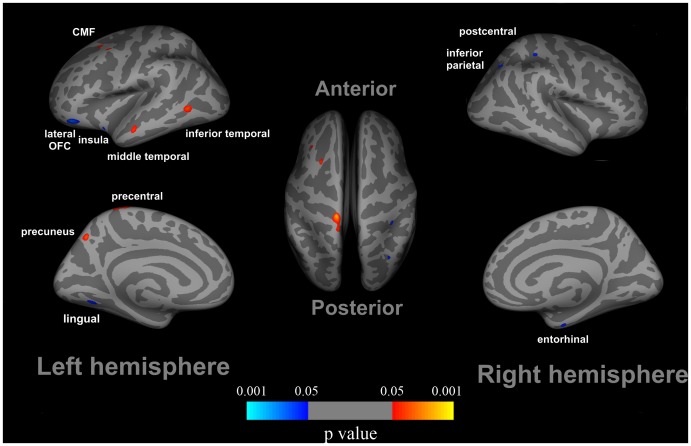
Cortical thickness differences in adolescents with online gaming addiction compared with healthy controls. After controlling for the Beck depression inventory-II (BDI) effect, increased cortical thickness was observed in several regions in late adolescence with online gaming addiction compared to healthy controls, i.e. the left precentral cortex, precuneus, middle frontal cortex, and inferior temporal and middle temporal cortices. In addition, reduced cortical thickness in the left lateral orbitofrontal cortex (OFC), insula cortex and lingual gyrus, along with the right postcentral gyrus, entorhinal cortex, and inferior parietal cortex were detected in late adolescence with online gaming addiction.

After controlling for age, education, gender, HAMA and BDI effects, there were several regions with significantly reduced cortical thickness in adolescents with online gaming addiction compared to healthy controls, which consisted of the left lateral OFC (−9%), insula cortex (−10%) and lingual gyrus (−10%), along with the right postcentral gyrus (−13%), entorhinal cortex (−13%), and inferior parietal cortex (−10%) (Figure 1). Additionally, the increased cortical thickness in the left precentral cortex (+14%), precuneus (+13%), middle frontal cortex (+10%), and inferior temporal (+11%) and middle temporal cortices (+11%) was observed in adolescents with online gaming addiction (Figure 1).

**Figure 2:**
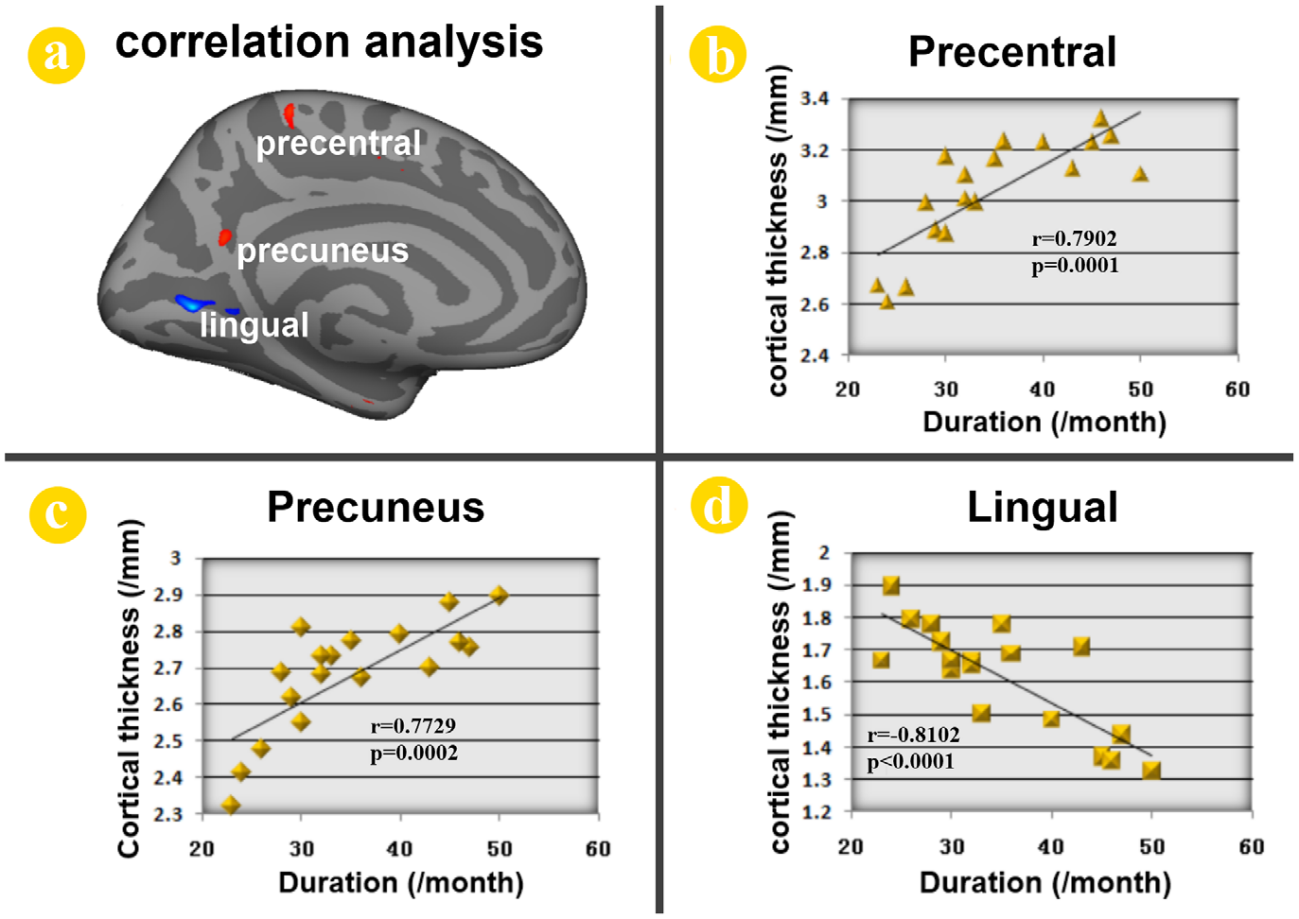
Correlation analysis results between cortical thickness and duration of online gaming addiction in late adolescence with online gaming addiction. The cortical thicknesses of the peak vertex within the clusters of the left precentral cortex (r = 0.7902, *p* = 0.0001) and precuneus (r = 0.7729, *p* = 0.0002) were found to be significantly positive correlated with the duration of online gaming addiction in late adolescence with online gaming addiction. The left lingual gyrus (r = −0.8102, *p*<0.0001) showed significantly negative correlation with the duration of online gaming addiction.

**Figure 3:**
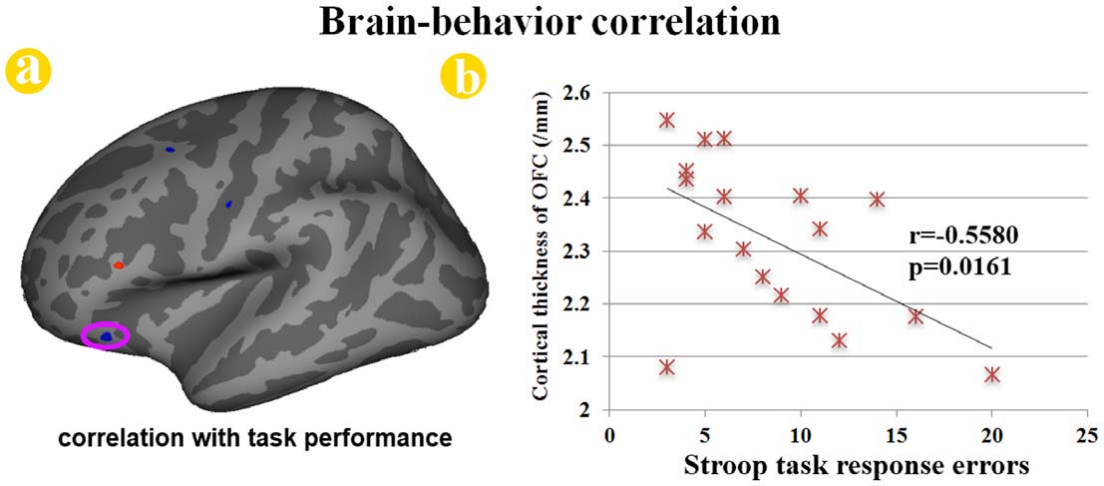
Correlation analysis results between cortical thickness and stroop task performances in adolescents with online gaming addiction. The cortical thickness of the peak vertex in the left orbitofrontal cortex (OFC) was significantly correlated with the number of errors during the incongruent condition among late adolescence with online gaming addiction (r = −0.5580, *p* = 0.0161).

The cortical thickness of the left precentral cortex (r = 0.7902, *p = *0.0001) and precuneus (r = 0.7729, *p = *0.0002) was positively correlated with the duration of addiction in adolescents with online gaming addiction (Figure 2). Only the left lingual gyrus (r = −0.8102, *p*<0.0001) showed a significantly negative correlation with the duration of online gaming addiction (Figure 2). Furthermore, the cortical thickness of the left OFC was inversely correlated with the number of errors during the incongruent condition among the adolescents with online gaming addiction (r = −0.5580, *p = *0.0161) (Figure 3).

## Discussion

IAD is a newly identified condition with loss of control over Internet use and has attracted worldwide attention [7], [9], [12], [13], [14], [15], [17]. According to the statistics from the China Youth Internet Association (announcement made on February 2, 2010), the incidence rate of IAD among Chinese urban youths is about 14% and 24 million in total (http://edu.qq.com/edunew/diaocha/2009wybg.htm). Furthermore, IAD has led to negative outcomes in the real social life and become the major source of adolescent crime in China [8], [12], [13], [17]. As a result, more attention should be paid to adolescents with the most popular subtype of IAD, i.e. online gaming addiction. Numerous functional imaging studies had detected the possible neural mechanisms of the online gaming addiction and suggested that it may share similar psychological and neurobiological abnormalities with addictive disorders with and without substance [20], [21], [22], [23]. Unfortunately, the cortical thickness abnormalities in the adolescents with online gaming addiction and the association between the cognitive control impairment and the cortical topography differences are not well known. Therefore, the purpose of the present study was to detect the cortical thickness abnormalities of the late adolescence with online gaming addiction. In addition, the color-word Stroop task performances were chosen as the behavioral assessment to investigate the functional implications of the cortical thickness differences. It is hoped that our findings can be used to develop novel imaging biomarkers that will enhance the understanding, diagnosis, and treatment of online gaming addiction.

The demographical information showed that online gaming addiction individuals spent 10.2±2.6 hours per day and 6.3±0.5 days per week on online gaming, which is significantly more than the normal controls (Table 1). Previous studies had revealed that the impaired cognitive control ability in the adolescents with online gaming addiction [29], [32]. To validate the impaired cognitive control ability in the adolescents with online gaming addiction, the color-word Stroop test was introduced in our study. Consistent with previous findings [29], the online gaming addiction individuals committed more errors than the control group during the incongruent condition. Our results demonstrated that the adolescents with online gaming addiction showed impaired cognitive control ability measured by the color-word Stroop test. Imaging results demonstrated that some brain regions associated with executive function showed decreased cortical thickness in online gaming addiction group, such as the left lateral OFC, insula cortex and entorhinal cortex; the others exhibited increased cortical thickness, such as the left precentral gyrus, precuneus and middle temporal cortex (Figure 1). Moreover, the correlation analysis demonstrated that the cortical thickness of several regions were significantly correlated with duration of addiction in adolescents with online gaming addiction (Figure 2), which were the left precentral gyrus, precuneus and the lingual gyrus. In addition, the reduced cortical thickness of the left OFC was correlated with the impaired cognitive control ability measured by the color-word Stroop task (Figure 3). The findings here demonstrated that there was a cumulative effect of online gaming addiction on the cortical thickness of these brain regions. The connection between the cortical thickness findings and the behavioral assessments could improve our understanding of the structural effects of online gaming addiction on the brain in the adolescents.

In the current study, we detected decreased cortical thickness in the left OFC (Figure 1). The OFC is highly involved in reward function and decision making [33] as evidenced by conclusions from previous drug addiction studies [34]. This area is an important part of the prefrontal cortex and has biological connections with crucial subcortical nodes associated with learning and reward, such as the basolateral amygdala and nucleus accumbens (NAc). By virtue of these connections, the OFC is uniquely positioned to use associative information to project into the future and to use the value of perceived or expected outcomes, and ultimately to guide decisions [33]. Additional lines of evidence from studies of addictions to substance showing structural abnormalities in the OFC concluded that the damages in the OFC are associated with an impaired ability in impulse control and decision-making [33]. Akin to a deficit in decision-making ability in substance addicts, adolescents with online gaming addiction also exhibited behaviors caused by degraded performance in decision-making, i.e. constant compulsive Internet-seeking behavior despite of their awareness of negative outcomes [12], [13], [35]. Moreover, the significant correlation between the cortical thickness of the OFC and the task performance during the color-word Stroop test was found in our current study (Figure 3). Previous addiction studies had revealed the association between Stroop interference and relative glucose metabolism in the OFC among cocaine-addicted subjects [36]. This brain-behavior relationship demonstrated that the abnormal structure of the OFC was associated with impaired executive function among adolescents with online gaming addiction. Our results provided more evidence for the structural changes in the OFC in adolescents with online gaming addiction.

We also detected reduced cortical thickness of the insula in adolescents with online gaming addiction, which is consistent with a former VBM study [24]. The insula was highlighted as a region integrating interoceptive states into conscious feelings and the decision-making process [37] and dysfunction of the insula may lead to abnormal decision making [38]. Recently, smokers with brain damage inclusive of the insula were found to be more prone to disruption of smoking addiction than smokers with brain damage exclusive of the insula [39]. The former subjects were characterized by the stronger ability to quit smoking immediately without relapse and the persistent urge to smoke. Our results suggested that the insula may be a critical neural substrate in online gaming addiction. Furthermore, thinner cortical thickness of the right inferior parietal lobule, postcentral gyrus and entorhinal cortex was also observed (Figure 1). Previous studies showed that the inferior parietal lobule was important to inhibitory control [40], cue-elicited cocaine craving [41] and gaming craving [22]. For the postcentral gyrus, a previous study also detected increased regional homogeneity in the postcentral gyrus in subjects with IAD [42]. In human brain tissue, dopamine receptor D4 (DRD4) was found in the entorhinal cortex [43] and the DRD4 receptor variants were associated with novelty seeking [44]. Adolescents exhibited novelty-seeking and risk-taking behaviors, which may be associated with progression from initial abuse to progressive addiction to several drugs [1]. Consistent with the previous VBM study [24], we detected reduced cortical thickness of the lingual gyrus in adolescents with online gaming addiction. Previous addiction studies revealed activation in the lingual gyrus during drug cue-related information processing [45], [46]. We provided scientific evidence for thinner cortical thickness of the inferior parietal lobule, the right postcentral gyrus and the entorhinal cortex in the current study (Figure 1). Evidently, more efforts are necessary to identify the accurate roles of these brain regions in online gaming addiction.

Apart form the decreased cortical thickness, increased cortical thickness of the left precuneus is identified in our study (Figure 1), which is associated with visual imagery, attention and memory retrievals [47]. Previous online gaming addiction study had revealed the activation of the precuneus for gaming cue reactivity [23]. Furthermore, the activation was correlated with gaming urge, craving and severity of online gaming addiction [23]. They suggested that the precuneus activates to process the gaming cue, integrate retrieved memory and contribute to cue-induced craving for online gaming [23]. In addition, increased cortical thicknesses of the inferior temporal cortex and middle frontal cortex were observed in the current study (Figure 1). The inferior temporal cortex [41] and the middle frontal cortex [48] have been engaged in craving induced by drug cues. Therefore, we suggested that the increased cortical thickness of the precuneus, the inferior temporal cortex and middle frontal cortex in online gaming addiction may be associated the craving of gaming cue.

The increased cortical thickness of the precentral cortex and the middle temporal cortex was also identified in the current study (Figure 1). Previous studies had established that the human brain has the ability to reshape itself to adjust to changes in the external environment or internal milieu [49], [50], [51], [52]. Adolescents with online gaming addiction spend a tremendous amount of time in on-line games for years becoming astonishingly skilled and accurate in mouse clicking and keyboard typing for better interaction of the player with the challenging environment during the game of the WOW. Given that the precentral cortex was mainly involved in planning and executing movements [53], [54], [55], [56] and the structural changes of the middle temporal cortex induced by training in previous VBM studies [51], [57], we suggest the cortical thickness changes in these areas may be associated the process of acquiring better playing skill upgrading from a “rookie” to an “advanced player”. However, the specific roles of the thicker regions in the adolescents with online gaming addiction require further investigation in future studies by employing a more comprehensive design.

Our study used a cross-sectional design and the question arises whether these differences were a consequence or precondition of online gaming addiction. Although, the correlation with the duration of online gaming addiction results may demonstrate that the cortical thickness changes of the brain regions in the present study were the consequences of online gaming addiction, this question could only be answered by investigating the temporal characteristics of experience-induced plasticity changes using a longitudinal design in the future. In addition, more cognitive measurements such as reward, craving and memory related tasks are needed to explain the findings of the present study.

### Conclusion

Our imaging results revealed decreased cortical thickness of the left lateral OFC, insula cortex, lingual gyrus, the right postcentral gyrus, entorhinal cortex, and inferior parietal cortex in adolescents with online gaming addiction; however, the cortical thicknesses of the left precentral cortex, precuneus, middle frontal cortex, inferior temporal and middle temporal cortices were increased. Correlation analysis demonstrated that the cortical thicknesses of the left precentral cortex, precuneus and lingual gyrus correlated with duration of online gaming addiction and the cortical thickness of the OFC correlated with the impaired task performance during the color-word Stroop task in adolescents with online gaming addiction. The findings in the current study suggested that the cortical thickness abnormalities of these regions may be implicated in the underlying pathophysiology of online gaming addiction.
